# The Mini Z Resident (Mini ReZ): Psychometric Assessment of a Brief Burnout Reduction Measure

**DOI:** 10.1007/s11606-022-07720-0

**Published:** 2022-07-26

**Authors:** M. Linzer, P. Shah, N. Nankivil, K. Cappelucci, S. Poplau, C. Sinsky

**Affiliations:** 1 Hennepin Healthcare, Minneapolis, MN USA; 2 American Medical Association, Chicago, IL USA

## INTRODUCTION

The Mini Z is a psychometrically sound measure of worklife and wellness in practicing clinicians.^1^ Measures in residents address slightly different issues and are moderately longer, potentially limiting response rates.^2^ We adapted the Mini Z for use in residents (the Mini ReZ), using the Mini Z core 10 questions and 5 additional questions reflecting domains identified by Trockel.^3^ Validating the new variation on the Mini Z would provide program directors the means to assess well-being within their programs (in collaboration with their residents), and determine actionable steps for improvement. The objective of this report is to demonstrate the validation of the Mini ReZ.

## METHODS

**Fig. 1:**
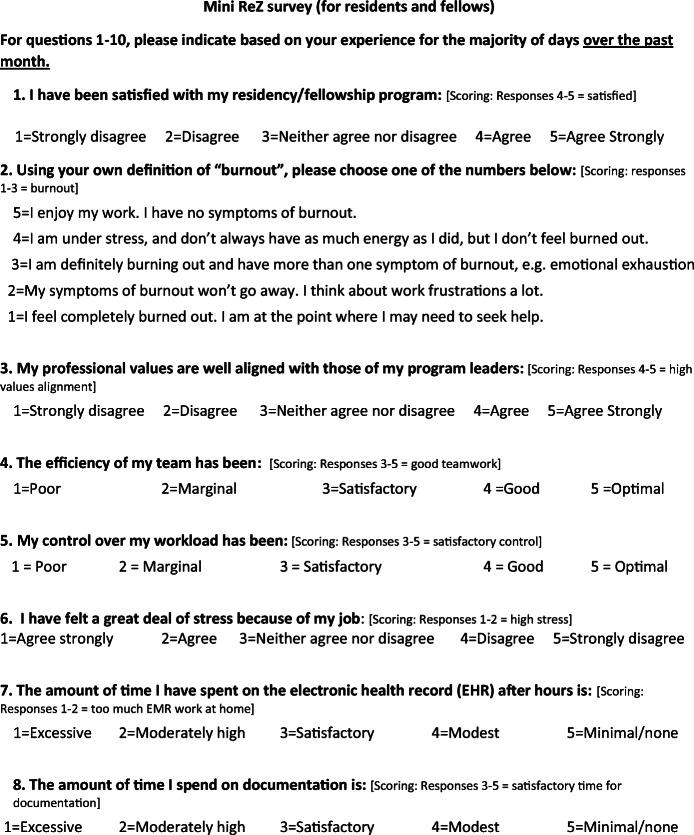

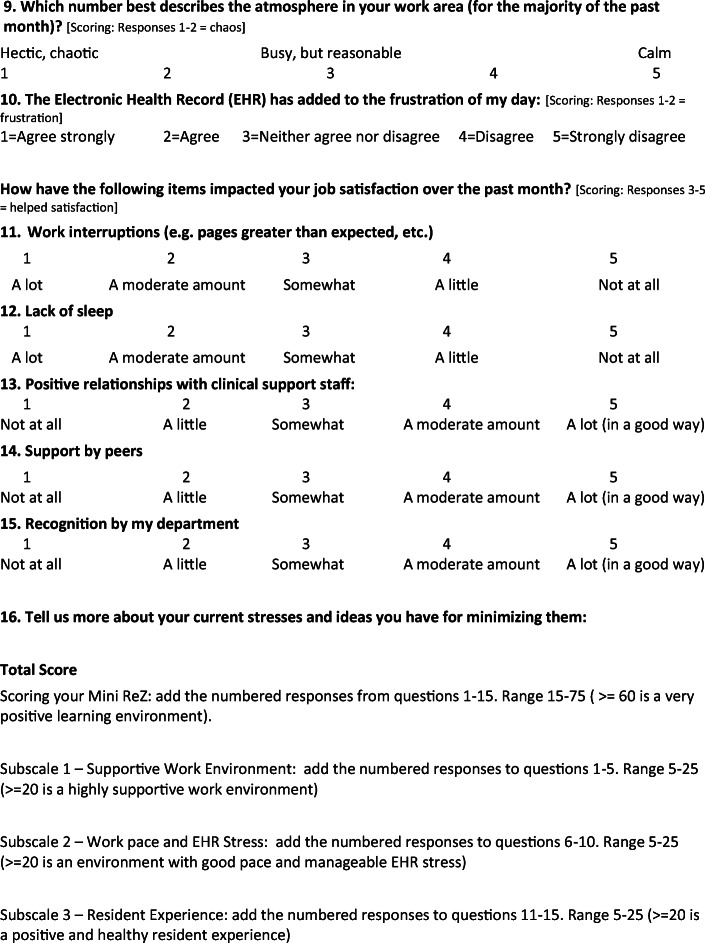
Mini ReZ with scoring. Items #1–10 adapted from Physician Worklife Survey (reference 4) and from MEMO study (Linzer M, Manwell LB, Williams ES et al. Working conditions in primary care: *Ann Intern Med*. 2009;151:28-36); questions #11–15 adapted from Trockel et al., reference 3. *The Mini Z was developed by Dr. Mark Linzer and team at Hennepin Healthcare, Minneapolis, MN. Mini Z survey tools can be used for research, program evaluation, and education without restriction. Permission for commercial or revenue-generating applications of the Mini Z must be obtained from Mark Linzer or Hennepin Healthcare Institute for Professional Worklife prior to use*: www.professionalworklife.com.

The American Medical Association (AMA) enrolled multiple US residencies in a worklife and wellness measurement program using a measure based on the Mini Z. The measure (Fig. 1) was distributed via a data platform through the AMA and Forward Health Group (Madison, WI) with data collected within the data lab. The core questions #1–10 (adapted in part from the Physician Worklife Survey)^4^ included satisfaction, stress, burnout, work control, chaos (work atmosphere), teamwork, values alignment, and electronic health record (EHR) challenges. A single-item 5-choice burnout measure, validated against emotional exhaustion in the Maslach Burnout Inventory, was scaled with the lower 3 choices representing “burnout.” Five additional questions (#11–15) reflected domains from Trockel’s work (interruptions, sleep impairment, support staff relationships, peer support, and program recognition). An intercorrelation matrix using Spearman’s method focused on relationships with burnout and a job satisfaction question from the Physician Worklife Survey.^4^ Correlations 0.35–0.69 represented modest to moderate correlations.^5^ An initial (derivation) set of correlations was performed for a 2019–2020 sample in 1792 US residents in 13 programs, with confirmatory correlations performed in 1097 residents in 12 programs (validation sample) surveyed in 2020–2021. Linear regressions adjusting for gender and program year assessed potential contributors to burnout.

## RESULTS

In the derivation sample, 43% of programs were in the Northeast, 25% Southeast, and 25% Midwest; 25% of respondents were PGY1’s, 22% PGY2’s, 30% PGY3’s, 13% PGY4’s, 5% PGY5’s, and 17% were fellows. Fifty-one percent were female and 46% were male. Thirty-five percent were persons of color. The largest number of residents was in internal medicine (16%); other programs with high representation included surgery (12%) and obstetrics and gynecology (6%). Correlation data are shown in Tables 1 and 2. Program satisfaction was most strongly associated with values alignment (*r* 0.67), teamwork (0.55), department recognition (0.50), work control (0.49), peer support (0.43), and positive staff relationships (0.42, all *p*’s < 0.0001). The burnout item provided good validation for the contributions of 4 of the 5 new single-item domains, including sleep impairment (*r* 0.54), program recognition (0.46), interruptions (0.43), and positive staff relationships (0.36, *p*’s < 0.0001). The only new item not reaching a 0.35 correlation was peer support (*r* 0.29). As in prior studies, burnout correlated significantly and meaningfully with work conditions, including values alignment, teamwork, work control, chaos, and time pressure (*r*’s 0.46–0.58). The validation sample showed comparable findings, with burnout related to most work conditions (values, teamwork, control, and chaos), and burnout and satisfaction both related to sleep impairment, department recognition, support staff relationships, peer support, and interruptions. Regressions confirmed strong contributions of dissatisfaction, stress, sleep impairment, and lack of recognition to burnout (*R* squared = 60% of burnout variance explained).

**Table 1:**
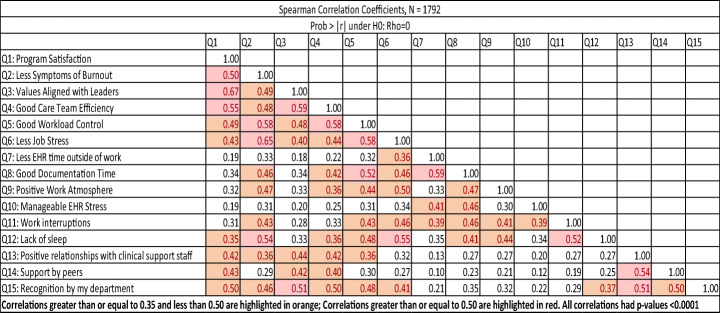
Intercorrelation Matrices. Mini ReZ National Resident Responses, 2019–2020 (Derivation Sample)

Correlations greater than or equal to 0.35 and less than 0.50 are highlighted in orange; correlations greater than or equal to 0.50 are highlighted in red. All correlations had *p* values < 0.0001. *EHR* electronic health record

**Table 2:**
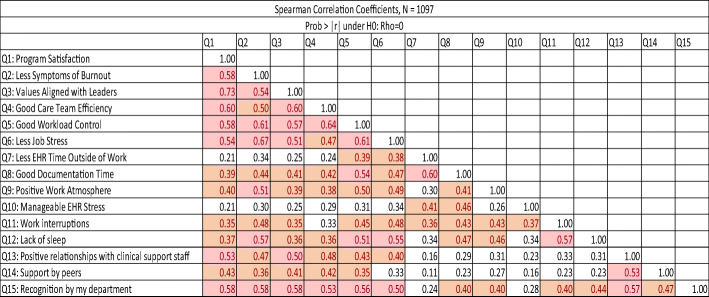
Intercorrelation Matrices. Mini ReZ National Resident Responses, 2020–2021 (Validation Sample)

Correlations greater than or equal to 0.35 and less than 0.50 are highlighted in orange; correlations greater than or equal to 0.50 are highlighted in red. All correlations had *p* values < 0.0001. *EHR* electronic health record

## DISCUSSION

Understanding resident worklife and wellness is currently of profound importance. High burnout and low job satisfaction are associated with depression, absenteeism, changing programs, or adverse personal outcomes.^6^ A brief measure, completed in a few minutes, could be valuable for programs wishing to sample their residents’ current state and build wellness-enhancing change together. These findings offer new insights into sources of job satisfaction for residents, including values alignment, teamwork, and recognition by their program, as well as validation of the importance of Mini Z work condition domains and 5 new items derived from well-tested surveys. Limitations include not having tested this against prior longer measures, a convenience sample of programs, and lack of information on the measure’s clinical utility. Use of the Mini ReZ to monitor resident worklife can be explored as a way to assess responses to residency, tailoring interventions to the findings.
